# Mixed-methods evaluation of the NHS Genomic Medicine Service for paediatric rare diseases: study protocol [version 1; peer review: 2 approved, 2 approved with reservations]

**DOI:** 10.3310/nihropenres.13236.1

**Published:** 2021-11-22

**Authors:** Celine Lewis, James Buchannan, Angus Clarke, Emma Clement, Bettina Friedrich, Jillian Hastings-Ward, Melissa Hill, Ruth Horn, Anneke M. Lucassen, Chris Patch, Alexandra Pickard, Lauren Roberts, Saskia C. Sanderson, Sarah L. Lewell, Cecilia Vindrola-Padros, Monica Lakhanpaul

**Affiliations:** 1 Population, Policy and Practice, UCL GOS Institute of Child Health, London, UK; 2 London North Genomic Laboratory Hub, Great Ormond Street Hospital for Children NHS Foundation Trust, London, UK; 3 Health Economics Research Centre, Nuffield Department of Population Health, University of Oxford,, Oxford, UK; 4 NIHR Oxford Biomedical Research Centre, Oxford, UK; 5 Division of Cancer and Genetics, Cardiff University School of Medicine, Cardiff, UK; 6 Clinical Genetics and Genomic Medicine, Great Ormond Street Hospital for Children NHS Foundation Trust, London, UK; 7 Independent Chair, Participant Panel at Genomics England, London, UK; 8 Genetics and Genomic Medicine, UCL GOS Institute of Child Health, London, UK; 9 The Ethox Centre and the Wellcome Centre for Ethics and Humanities, Department of Population Health, University of Oxford, Oxford, UK; 10 Clinical Ethics and Law, Faculty of Medicine, University of Southampton, Southampton, UK; 11 Genomics England, Queen Mary University of London, London, UK; 12 Counselling, Society and Ethics Research, Wellcome Genome Campus, Cambridge, UK; 13 Genomics Unit Specialised Commissioning, NHS England, London, UK; 14 Genetic Alliance UK, London, UK; 15 Our Future Health, London, UK; 16 Unique – the Rare Chromosome Disorder Support Group, Oxted, UK; 17 Department of Targeted Intervention and Rapid Research Evaluation and Appraisal Lab (RREAL),, University College London, London, UK

**Keywords:** genomics, genomic medicine service, rare disease, paediatric, protocol, mixed methods

## Abstract

**Background:**

A new nationally commissioned NHS England Genomic Medicine Service (GMS) was recently established to deliver genomic testing with equity of access for patients affected by rare diseases and cancer. The overarching aim of this research is to evaluate the implementation of the GMS during its early years, identify barriers and enablers to successful implementation, and provide recommendations for practice. The focus will be on the use of genomic testing for paediatric rare diseases.

**Methods:**

This will be a four-year mixed-methods research programme using clinic observations, interviews and surveys. Study 1 consists of qualitative interviews with designers/implementers of the GMS in Year 1 of the research programme, along with documentary analysis to understand the intended outcomes for the Service. These will be revisited in Year 4 to compare intended outcomes with what happened in practice, and to identify barriers and facilitators that were encountered along the way. Study 2 consists of clinic observations (pre-test counselling and results disclosure) to examine the interaction between health professionals and parents, along with follow-up interviews with both after each observation. Study 3 consists of a longitudinal survey with parents at two timepoints (time of testing and 12 months post-results) along with follow-up interviews, to examine parent-reported experiences and outcomes. Study 4 consists of qualitative interviews and a cross-sectional survey with medical specialists to identify preparedness, facilitators and challenges to mainstreaming genomic testing. The use of theory-based and prespecified constructs will help generalise the findings and enable integration across the various sub-studies.

**Dissemination:**

We will disseminate our results to policymakers as findings emerge, so any suggested changes to service provision can be considered in a timely manner. A workshop with key stakeholders will be held in Year 4 to develop and agree a set of recommendations for practice.

## Introduction

### Context

In October 2018, a new nationally commissioned Genomic Medicine Service (GMS) was established by NHS England. This service, built around seven Genomic Laboratory Hubs (GLHs), aims to deliver consolidated, state of the art, high throughput and high-quality genomic testing (including both genome and exome sequencing) with equity of access for patients affected by rare diseases and cancer^1^. The GMS capitalises on the infrastructure and learning from the 100,000 Genomes Project, a world-leading initiative set up in England in 2015 with the explicit aim of embedding genomic medicine into clinical care to improve diagnosis and management of patients affected by selected rare and inherited diseases and cancer^2^. The NHS will be the first national healthcare system in the world to offer whole genome sequencing as part of routine care.

The overall goal of the GMS is that from 2020, and by 2025, genomic medicine will be embedded in multiple clinical pathways in routine care, where appropriate, and linked to a broader NHS long term plan of sequencing 500,000 whole genomes for patients with certain rare diseases and cancers, incorporating the latest genomics advances into routine healthcare to improve diagnosis, stratification and treatment of illness, and supporting research and innovation^3^. Ultimately, the aim is that by 2025, genomic technologies will be a fundamental component of medical training, and there will be a new taxonomy of medicine based on the underlying drivers of disease^4^.

### Mainstreaming genomics for rare disease diagnosis

Genome sequencing will be available as a first-line test for some rare and undiagnosed diseases, for example individuals with ultra-rare disorders or atypical manifestations of recognised monogenic disorders. In addition, certain tests specified in the new NHS England Genetic Test Directory can be ordered by medically qualified individuals specialised in a sub-discipline other than genetics (referred to hereon in as ‘medical specialists’), in both primary and secondary care, thus ‘mainstreaming’ genomics^1^. For example, in primary care, a general practitioner could order a cystic fibrosis carrier test, and in secondary care a neurologist or paediatrician could order genome sequencing for a patient with intellectual disability.

### How will the NHS GMS impact health professionals?

The significant changes in the way testing is offered will impact across medical specialities and require the roles of both medical and genetic specialists to evolve^5^. Widespread implementation and ‘mainstreaming’ of genomic medicine will depend on health professionals’ perceptions of the usability and value of the technology in day-to-day practice, however some of these professionals are sceptical of the positive impact genomic medicine will have on patient care^6,7^. Studies have shown that many health professionals have limited genetics training and may be unprepared to conduct pre- and post-test counselling including interpreting test results and consenting/returning additional findings^8,9^. Concerns also exist around lack of access to genetic professionals^10^ as well as the challenges of interpreting uncertain results and managing patients’ expectations about genome sequencing^11^. To ensure the successful transition of genomics from a specialist service to a mainstream service, thoughtful planning and procedures are required to prepare the workforce. This includes: training in genomics for healthcare professionals outside of clinical genetics including interpreting and returning genomic data back to patients; clear pathways for which tests to order for which indications; educational initiatives to ensure healthcare professionals taking consent feel equipped to do so; and increased interaction between genetic and medical specialists to support the delivery of testing outside the clinical genetics specialty^5^.

### Preparation for genomic testing in the NHS GMS

Over the past few years, several initiatives have been implemented to prepare the workforce for genomic testing. In 2014, Health Education England (HEE) launched a four-year £20 million Genomics Education Programme (GEP) to ensure that the NHS workforce has the knowledge, skills and experience to keep the United Kingdom (UK) at the heart of the genomics revolution in healthcare^3^. Other initiatives include a Masters in Genomic Medicine delivered by seven leading higher educational institutions; ‘genomics roadshows’ where genetic specialists have visited a wide range of clinical disciplines in hospitals to highlight genomics and how it can improve patient care; and a genomics toolkit developed by the Royal College of General Practitioners in partnership with the GEP to explain how genomic medicine impacts primary care^3,12,13^. However, the reach and utility of these resources have yet to be examined, and the informatics infrastructure including sample collection pathways and results delivery processes have yet to be finalised and tested.

To ensure patients and families are fully prepared for genomic testing in the NHS GMS, NHS England have prepared a range of patient-facing and online resources^14–16^, as well as a ‘record of discussion’ form which will be used in the clinical pathway to record parents’ (of children unable to consent themselves) and patients’ test and research decisions^17^. Genomics England has developed information specifically to support people making decisions about participating in research which will be done on a voluntary basis and consented separately from genomic testing for clinical care^18^. However, we do not yet know what patients’ and parents’ attitudes, understanding and experiences of genomic testing within a purely clinical context will be, whether they feel they have made an informed decision to undergo sequencing, what proportion will consent to donating their (or their child’s) data for research purposes (and if not, why not), and whether they are satisfied with the process overall.

The first few years of the NHS GMS is an ideal opportunity for which to evaluate the implementation, service and patient outcomes of genomic testing in a clinical setting. It will enable us to make comparisons with the hybrid research-clinical context of the 100,000 Genomes Project where much research has already taken place^7,19–24^.

### How will the NHS GMS impact parents and children?

The NHS GMS is set to have a profound impact on the management and diagnosis of children with rare diseases in the NHS. The majority (50–75%) of rare diseases affect children^25^ and in the past it has taken on average six years for a rare disease to be diagnosed, during which time patients are likely to have undergone extensive medical testing^26,27^. Genomic sequencing has the potential to reduce this ‘diagnostic odyssey’ for some patients with rare diseases and their families. The diagnostic yield of genomic sequencing in previously unsolved paediatric cases is already around 40–50% and may increase as knowledge grows^28^. For children with a rare condition, a diagnosis can enable access to disease specific screening or treatments, provide a clearer prognosis and information about recurrence risk, enable parents to make contact with other parents, and facilitate access to social and educational support^29^. Psychological benefits for parents can include relief from guilt, understanding the origin of the child’s condition, validation in terms of offering legitimacy for the child’s behaviour and/or appearance, and ability to connect with others through support groups^30^.

Previous research on patients and parents experiences of genomic testing, conducted during the 100,000 Genomes Project, highlighted that the majority were satisfied with the consenting process, felt they had made an informed decision to take part, and had largely positive attitudes towards sequencing, although concerns existed around data sharing and access, and the potential emotional impact of the results^19^. Whilst participants generally understood what is involved in genome sequencing, the purpose and the benefits, there were misunderstandings around the limitations and associated uncertainties^24^. For example, only around 70% of participants correctly understood that they may not receive any informative results about their child’s condition from whole genome sequencing^24^. Reports of parents misinterpreting or overestimating the utility of findings from genomic testing have been cited elsewhere^31,32^, and the importance of managing patient expectations to avoid disappointment or decisional regret has been raised by genetic specialists^33,34^. Research focused on whether and how health professionals are managing parental expectations of genomic testing in the NHS GMS would therefore be of value.

Whilst evidence has begun to emerge about the clinical effectiveness of genomic testing (e.g. changes in clinical management, amended treatment plans) for patients from different condition groups^35,36^, for example those having rapid genomic testing in the neonatal setting^37^ or those with developmental disorders^38^, we still have limited data on the psychosocial and behavioural impact of disclosing genomic results to parents, including whether and how the impact differs amongst different patient populations^39^. There is some evidence to suggest that parents of children with a known disease may be more prone to negative test-related psychological experiences following genomic testing than other population groups^40^. Results from the 100,000 Genomes Project indicated that some participants and in particular parents experienced distress and uncertainty following receipt of sequencing results. Similar findings have been reported elsewhere, with parents receiving exome sequencing results reporting feelings of frustration and isolation from the lack of available information about the condition^41^ as well as loss of hope for recovery^42^. However, this research is still in its infancy, and further research is essential to gain a more nuanced and complete understanding of the psychosocial and behavioural impact of genomic testing.

## Protocol

### Research aims and objectives

This paper outlines a four-year mixed-methods research programme using observations, interviews and surveys to evaluate the NHS GMS for the diagnosis of paediatric rare diseases. Research will be conducted with key stakeholders designing and implementing the GMS, as well as health professionals (genetic and medical specialists) and parents of patients undergoing genomic testing to examine the intentions, experiences and outcomes of the new service^43^.

The aims are to:

1. Identify the **resources, activities and intended and actual outcomes** of the NHS GMS; identify **any potential barriers to achieving the intended outcomes** during the early years of the Service (2022–25);
2. Understand the processes and practices taking place by **examining the interactions between health professionals and parents/patients** during pre-test counselling and results delivery appointments;
3. Examine the **experiences and outcomes** of genomic testing that parents report **over time**;
4. Identify the **preparedness and experiences** of medical specialists involved in delivering genomic medicine in main-stream NHS care in the first few years of the Service, and identify elements which make this easier or more difficult.

The findings from the research will be shared with NHS England and NHS Improvement contemporaneously to continue to drive improvements in the Service and develop recommendations for practice.

### Methods and analysis

#### Research approach and conceptual framework

We will conduct a mixed-methods research programme, employing qualitative and quantitative approaches to provide a richer, deeper insight into the topic area, generating more knowledge, and increasing the validity of the findings^44^. We will work within a pragmatist paradigm in order to seek functional knowledge and produce positive change in clinical practice^45^. Pragmatism refers to a worldview that focuses on “what works” rather than what might be considered absolutely and objectively “true” or “real.”^46^.

We will use a theory-driven approach to understand how the NHS GMS is being implemented as well as to evaluate the outcomes from the Service. The Consolidated Framework for Implementation Research (CFIR)^47^ will be used as an explanatory framework to systematically assess the contextual factors including barriers and facilitators that influence implementation and adoption, and has been used previously to evaluate the implementation of genomic medicine^48–51^. The framework provides a taxonomy of operationally defined constructs that are likely to influence implementation of complex programs, organised into five major domains: 1) Intervention Characteristics; 2) Outer Setting; 3) Inner Setting; 4) Characteristics of Individuals; and 5) Process. We will evaluate implementation outcomes according to Proctor’s taxonomy, which comprises eight major domains - acceptability, adoption, appropriateness, feasibility, fidelity, implementation cost, penetration, and sustainability^43^. In order to understand the patient/parent perspective, we will use a number of patient reported outcome measures including decisional conflict and regret, patient empowerment and satisfaction. The use of theory-based and pre-specified constructs will help to generalise the findings and enable integration across the various sub-studies, enabling us to build a stronger evidence base.

#### Study design overview

**Figure 1:**
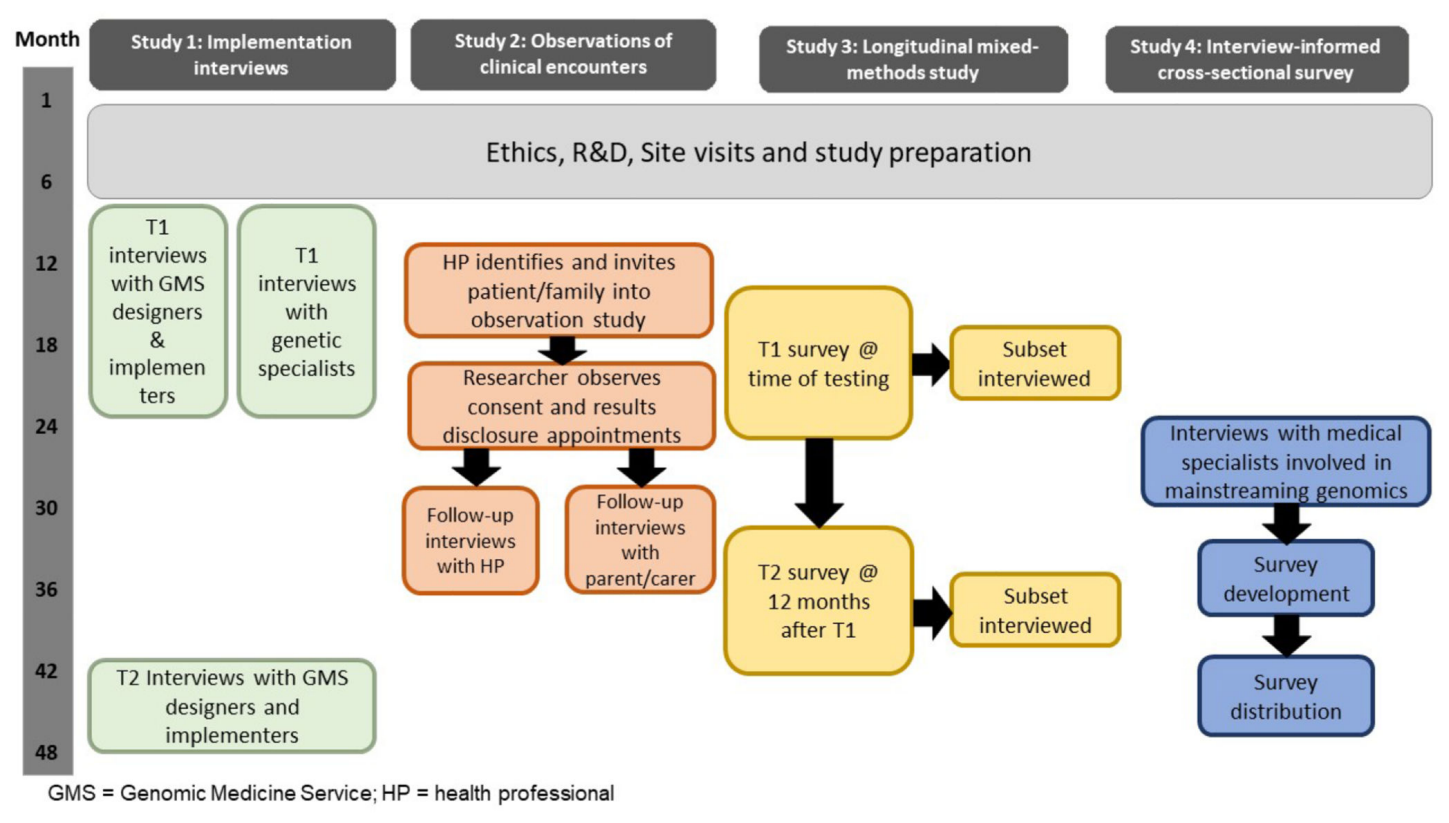
Overview of study timelines.

Using an implementation science approach, we will conduct an interview study with key stakeholders from organisations tasked with designing and implementing the NHS GMS (e.g. Genomics England, NHS England) along with desk-based documentary research to examine the initial programme theory (resources, activities and intended outcomes) underlying the NHS GMS. These will be compared with the actual outcomes in Year 4 in terms of effectiveness, adoption, fidelity, acceptability and uptake (Aim 1 – Study 1). We will also conduct interviews with genetic specialists tasked with delivering the NHS GMS to understand how they are experiencing the implementation (Aim 1 – Study 1). We will conduct observations of clinical encounters to examine the interaction between healthcare professionals and parents, alongside interviews with the professionals and parents to understand the processes and practices taking place when consenting for and returning genomic test results (Aim 2 – Study 2). We will conduct a longitudinal survey along with follow-up interviews to examine parent-reported experiences and outcomes from genomic testing (Aim 3 – Study 3), and we will conduct interviews followed by a cross-sectional survey with medical specialists in Year 4 to identify the retrospective preparedness, experiences and challenges to delivering genomic testing in the first few years of the NHS GMS (Aim 4 – Study 4). See Figure 1 for overview of study design and timelines.

#### Patient and public involvement

The research programme has been co-designed with parents of children with rare diseases as well as patient advocates and key stakeholders. At the time of drafting the funding application, input was sought from Genetic Alliance UK, Rare Disease UK and SWAN UK (Syndromes without a name) to identify the key research questions and discuss study design, ensuring the design facilitated patient-orientated outcomes. CL also spoke with the Chair of the 100,000 Genomes Project Participant Panel as well as a parent of a child with a rare undiagnosed condition, to ensure the study design would capture what was important to parents.

Following approval for funding, an Advisory Team was set up, which includes three parents of children with (previously) undiagnosed rare conditions and two patient advocates from the support groups SWAN UK and Unique: The Rare Chromosome and Gene Disorders Support Group. They have inputted into the study aims and objectives, reviewed and revised patient-facing documents including participant information sheets and topic guides (Studies 2 and 3), informed the selection of validated measures for a longitudinal survey study (Study 3) and commented on wording and answerability. Emergent findings will be shared with the advisory team throughout the study, and they will support the development of recommendations for policy and practice, ensuring that they are feasible and appropriate. Policy options will be evaluated using the APEASE framework (Acceptability, Practicability, Effectiveness, Affordability, Side-Effects, Equity)^52^. The APEASE criteria are a set of criteria used to make context-based decisions on intervention content and delivery. The advisory team will advise on plain language summaries and video abstracts to facilitate dissemination of the study findings to participants and relevant wider patient communities, and will be invited to co-author manuscripts. Parent participants will be reimbursed for their involvement in the project, in line with the NIHR Centre for Engagement and Dissemination’s payment policy^53^.

#### Study setting

Recruitment of participants will take place across seven NHS Trusts located across England. Sites have been selected to facilitate a diverse ethnic mix of participants as well as North vs South and urban vs rural settings. At each participating site, a health professional from the genetics department will act as local Principal Investigator (PI) for the study, however we will work closely with departments outside of clinical genetics who are delivering genomic testing to examine the issue of mainstreaming. Regular meetings will be held with the participating site and clinical staff to discuss any recruitment issues.

#### Participants

There will be two separate but parallel cohorts in this project; 1) parents of children (<16 years) with rare diseases undergoing genomic testing, who are making decisions on behalf of their children and may themselves be undergoing testing to help identify or interpret the results, and 2) health professionals (including genetic and other medical specialists), policy-makers, commissioners and organisational decision-makers who are delivering the GMS. By examining the way in which key stakeholders (parents, health professionals, policy-makers, decision-makers) perceive, experience and behave in the GMS, we will ensure that the findings and subsequent recommendations around the implementation of genomics into mainstream clinical practice are grounded in first-hand experiences.

For Study 2, In order to accommodate participants that do not speak/have limited English, we will translate the participant information sheet and consent form into those languages identified as being commonly spoken by parents attending genetic services (e.g. Gujarati, Bengali, Urdu, Polish, Punjabi). Regular dialogue between the research team and the PIs from participating sites will take place to monitor this. It will not be possible to translate the survey (Study 3) into these languages as the included measures have not been validated in these languages.

## Detailed study plan

**Figure 2:**
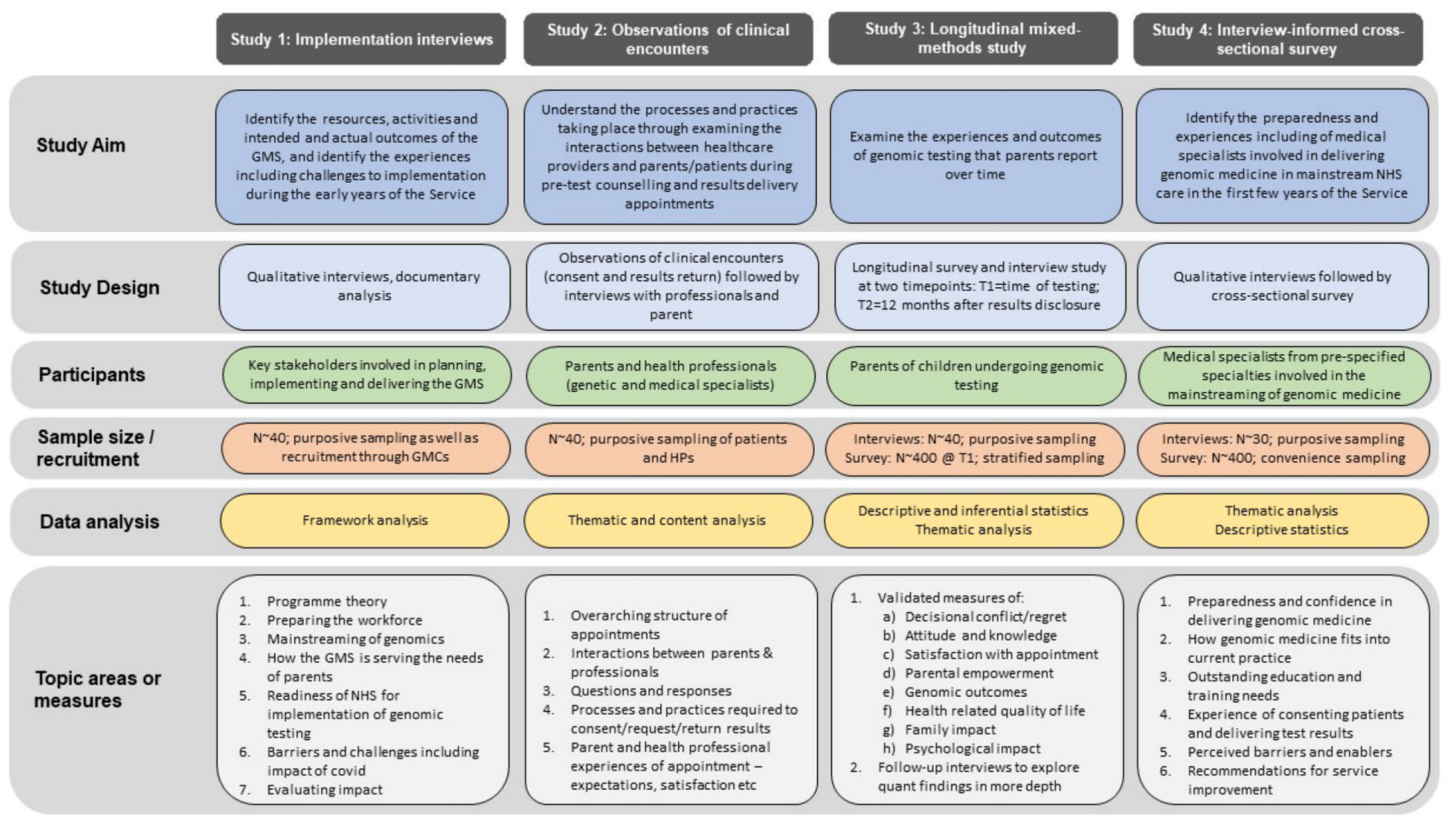
Study design.

An overview of the study plan is provided in Figure 2.

### Study 1: Implementation interviews with key stakeholders (Years 1 and 4)

#### Study design

Implementation science, the systematic study of methods that support the application of research findings and other evidence-based knowledge into policy and practice, is increasingly being seen as playing a critical role health services research^47,54^. Previous research on new interventions has highlighted that as well as assessing outcomes, it is valuable to look at the process of the intervention as this can shed light on the mechanisms responsible for whether and how successful it is 55. This formative work can also enable researchers to suggest ways to answer questions about how interventions might be adapted and respond to change in order to produce positive outcomes^56^.

In Year 1 of the study, qualitative interviews will be conducted with key stakeholders involved at a national level in planning and implementing the new Service (from organisations such as NHS England, Health Education England, Genomics England as well as ‘genomic champions’^57^ from different clinical specialties). In addition, documentary evidence such as policy documents, journal articles and meeting presentations will be collated and analysed. This will enable us to identify the initial programme theory underpinning the GMS and to identify the underlying assumptions about how the GMS is expected to work to achieve its expected outcomes. A logic model (a visual representation of the theory), which describes the resources and activities (inputs) and intended outputs, outcomes and impact will be developed^58^. This will form the foundation for our understanding of what was intended for the GMS (the GMS ‘blueprint’). In Year 4, we will conduct further interviews and documentary analysis to understand if the programme theory has changed over time, if the programme as planned is different to the programme as performed (‘fidelity to the model’), and the factors that have acted as barriers and facilitators in implementation.

Alongside interviews with designers and implementers, we will also conduct interviews with genetic specialists embedding and delivering genomic services including directors of GMSs and GLHs. The aim of these interviews will be to understand how they are experiencing the implementation of the GMS during its first years including: the processes and procedures that have been put in place to deliver the Service; any individual and organisational adaptations that have been made; and identify any challenges professionals have faced when implementing the Service. Interview guides will be informed by the CFIR and Proctor’s taxonomy, the existing literature and the key research questions.

#### Data analysis

Data will be analysed using framework analysis^59^. This is an approach that facilitates identification of key themes as well as commonalities and differences in the data through comparison across as well as within cases. A codebook will be developed to facilitate team-based analysis which will be facilitated using NVivo software^60^. The first step will consist of a deductive analysis, where data are coded according to the CFIR domains and constructs. This will be followed by an inductive analysis, to allow for any new themes or unexpected findings. The same codebook will be applied to the analysis of both sets of interviews as well as the documentary evidence to enable cross-referencing and comparisons across the data.

#### Recruitment and sample size

Designers and implementers identified initially by the advisory team, will be purposively sampled and invited for interview. In addition, we will use snowball sampling to ensure that key players not known to the advisory board are invited^61^. Genetic specialists will be recruited from across the seven GLHs. Informed consent along with participant information will be collected prior to interview. Interviews will continue until saturation is reached and, alongside documentary evidence, the initial programme theory has been identified. We anticipate this will be around 10–20 interviews with designers and implementers, and 14 interviews with genetic specialists (two per GLH).

### Study 2: Observations of clinical encounters (Years 2–3)

#### Study design

We will conduct direct observations (including audio and/or video-recordings) of clinical encounters (clinical pre-test counselling appointments as well as results delivery appointments) involving patients (children) and families undergoing genomic testing. A key benefit of observations is that they take place in natural settings that are the natural loci of activity^62^. As such, they will help us to understand consistencies and variations in the overarching structure of the appointments, evaluate the interactions between patient/parents and health professionals (including the information exchange and the questions and responses), and gain insight into the communication techniques that are employed by both parties. In addition, the observations will offer insight into the various processes and practices required in order that health professionals can request genomic tests (patient choice forms, uploaded test requests etc). Observations of clinic appointment were previously conducted during the 100,000 Genomes Project and yielded valuable data^20^. A structured observation guide using pre-determined categories identified through this previous work (e.g. checklist of particular topics, interactions between the professional and the [child] patient, notable non-verbal behaviours, paperwork and administrative aspects etc) will be used to standardise the observation and inform follow-up interview questions.

Following each observation there will be an immediate de-brief interview with the professional and an interview with parents 1–2 weeks later. The topic guides will focus on views and feedback related to the content of the appointment, their expectations and implicit goals from the interaction, and moderating factors that may have hampered or contributed to the success of the appointment. This will allow for comparison across the three data sources (interview recording, professional and parent interviews). Pairing observations/audio-recordings with interviews is valuable because this may reveal inconsistencies between participants’ responses to interview questions and what they actually do in practice^63^.

#### Data analysis

The analysis will be conducted from an interactionist perspective^64^ using concepts drawn from content analysis^65^ and thematic analysis^66^, facilitated using Nvivo^60^. Data from the different sources will be given equal weighting and integrated at the data analysis stage, to explore the appointment from multiple perspectives.

#### Recruitment and sample size

Eligible participants will be parents, carers or other family members of children undergoing genomic testing for rare disease diagnosis. Non-English speaking families will be eligible to participate provided the translator is able to translate the participant information sheet and consent form. Potential participants (health professionals and parents) will be purposively sampled to ensure variation in 1) condition type, 2) who is conducting the appointment (genetic or medical specialist), 3) result (diagnostic result, nofinding result and inconclusive result), and 4) site. By including seven sites from regionally diverse parts of the country, we hope to include parents who vary in terms of educational and socioeconomic status as well as ethnicity. People from minority ethnic groups may experience a higher incidence and prevalence of rare diseases than the general population for a variety of reasons, genetic and otherwise^67^. In addition, people from minority ethnic groups and other underserved populations are likely to experience even greater barriers to screening, diagnosis, and treatment of rare diseases than for common conditions due to a variety of cultural, socioeconomic, environmental and other factors^67^.

Observations of a given professional will take place no more than once to ensure maximum variation in participants. We will aim to observe ~20 consent appointments and similarly ~20 results return appointments (with different participants to those observed during the consent process) in line with previous research^20^.

### Study 3: Longitudinal mixed methods study of parent-reported experiences and outcomes from genomic testing (Years 2–4)

#### Study design

Surveys and interviews will be conducted with parents of children with rare diseases to evaluate parent-reported experiences and outcome from genomic testing in the GMS.

An online survey will be administered at two time-points; after pre-test counselling (T1) and approximately 12 months after results-disclosure (T2). Our primary outcome measure is decisional regret at T2, as measured on the validated Decisional Regret scale^68^. In particular, we will compare whether decisional regret differs between parents of patients who get a diagnostic result compared with those that get a no primary findings result. Whilst there is limited data on the psychological effects of disclosing genomic sequencing results to parents of paediatric patients, a number of studies have shown that a subset of parents may be likely to experience decisional regret^39,69^ and that regret may be linked to parents interpretation of the child’s result as negative and of frustration with uncertain results^69^. Key research questions will be whether there is a difference in levels of decisional regret depending on result status, and whether there is a difference in levels of decisional regret depending on clinical indication.

Secondary outcomes include knowledge^70^, attitudes^24^ (adapted from previous research^71^), self-reported informed decision-making^24^, decisional conflict^72^, generalised anxiety^73^ parental empowerment^74^, health-related quality of life of the child^75^, family impact^76^, psychological impact^77^ and satisfaction with appointment^78^ (see Table 1). We will explore whether parent characteristics e.g. education, ethnicity, and personality traits e.g. intolerance for uncertainty^79^ and resilience^80^ are associated with particular psychological outcomes.

To complement the quantitative results, a subset of survey responders will also be invited for a qualitative interview. Interviews will focus on parents’ expectations, experiences of and satisfaction with the consent appointment/return of results, perception of care received, clinical, behavioural, and psychosocial impact of the result, unexpected outcomes, and recommendations for service improvement.

#### Data analysis

This mixed methods study will use a concurrent design with quantitative and qualitative data collected in parallel and given equal status, the purpose being to seek a more complete understanding using complementary methods^81^. Qualitative and quantitative data will be analysed separately and integrated at the point of interpretation. Each set of findings will be brought together into one explanatory framework^81^.

For the quantitative data, frequencies, means and standard deviations will be calculated, and descriptive statistics will be reported. Correlations and comparative analyses will be conducted to identify changes over time (between T1 and T2). We will conduct correlations and t-tests (normally distributed variables) or Spearman’s rank correlation and paired-Wilcoxon signed ranks tests (non-normally distributed variables) to examine bivariate associations between the primary dependent variables and participant characteristics (e.g. gender, age, employment, education, ethnicity, resilience etc). Analysis will be facilitated using SPSS software^82^.

Qualitative data will be analysed using codebook thematic analysis^83^. This is a flexible analytic method where a code-book with both deductive (guided by theory and/or previous literature) and inductive (emerging from the text) codes are used to guide data coding, and allows for multiple researchers to systematically code the text. Codes are then collated to form sub-themes and themes, patterns of meaning anchored by a shared idea or concept. Analysis will be facilitated using Nvivo^60^.

#### Recruitment and sample size

Survey participants will be recruited from across the seven participating recruitment sites with the aim of recruiting participants from different geographical, ethnic and socio-economic backgrounds. We will recruit participants whose children have different clinical indications (e.g. neurological including intellectual disability, developmental delay and/or epilepsy, renal, cardiac) as well as those with single system (e.g. a heart defect) and multisystem (e.g. a kidney and heart defect) to facilitate exploratory comparisons across disease groups. Interview participants will be selectively sampled for maximum variation in terms of condition, result (diagnostic, negative or inconclusive) and socio-demographic factors. Where both parents attended the initial genomic testing appointment, only one parent per family (‘the main care-giver’) will be invited to complete the survey in order to avoid non-independence of results as family members may influence each other’s responses.

To compare decision regret between those parents of patients who received a diagnostic result and those who don’t, a minimum of 67 participants are required in both groups to achieve a medium effect size (0.5) with an 80% power level. As diagnostic rates using genomic testing are currently around 40% when trio-based analysis is performed^28^, a minimum of 168 participants is required. To account for drop-out between the T1 an T2 survey, which was around 50% in previous research^24^, we will aim to recruit around 400 participants at T1.

Recruitment for interviews will continue until saturation is reached, however we aim to interview around 20–30 parents at both timepoints. This is in line with previous qualitative interview studies exploring parental experiences of genomic testing^19^. Ideally, the same parents will take part in interviews across the two timepoints to examine the patient journey including parent expectations and outcomes.

### Study 4: Interview informed cross-sectional survey with medical specialists (Year 4)

#### Study design

Cross-sectional qualitative interviews will be conducted with non-genetic medical specialists to explore their experiences of current genomic practice. The topic guide will be informed through the CFIR and Proctor’s taxonomy, and include questions to assess their preparedness for delivering genomic medicine (consenting patients and delivering results), how genomic medicine fits into their current practice, outstanding education and training needs, interaction with genetic specialists, whether the nature of their clinical interactions with patients and families has changed over time, and to identify policy and/or service provider factors affecting ‘mainstream’ implementation of genomic medicine, including emergent enablers and barriers. The findings from the interviews will be used to inform the development of an anonymous cross-sectional online survey, which will also use validated measures to assess concepts such as acceptability^84^, feasibility^84^, implementation leadership support^85^ and organisational change expectations^86^. Survey data will provide evidence to policy makers about the effectiveness of mainstreaming.

#### Data analysis

Qualitative data analysis will be thematically coded^83^ using a codebook approach. The first step will consist of a deductive analysis, where data are mapped on to the CFIR and Proctor domains and constructs. This will be followed by an inductive analysis, where new themes or unexpected findings are elicited through coding and categorising. Quantitative data will be analysed using descriptive statistics.

#### Recruitment and sample size

Medical specialists from a chosen set of four to five specialties who are (expected to be) involved in the mainstreaming of genomic medicine (e.g. community paediatricians, paediatricians, paediatric neurologists, paediatric cardiologists) from across the seven recruiting sites will be purposively sampled for interview. Interviews will be conducted until saturation is reached, but we expect to interview around 5–10 per speciality in line with previous qualitative research looking at health professionals’ experiences of offering genomic testing^7^.

The online survey will be administered with links circulated across the seven participating sites as well as via health professional associations (e.g. Royal College of Paediatrics and Child Health). As this is a single topic community study, we will aim to recruit around 400 participants (around 100 for each medical speciality).

### Data synthesis and interpretation from all studies

The findings from the four studies will be analysed separately. However, at the end of the study we will integrate the data to draw overarching conclusions about service provision. Summary tables will be developed to identify context-specific barriers and facilitators (or suggested changes) to implementation. To enhance trustworthiness, qualitative data analysis will be conducted by multiple researchers. In addition, the advisory team, including the PPI group will support the interpretation of the data and ensure credibility of the data analysis. Further refinement of recommendations for practice will be developed at a workshop in Year 4 with key stakeholders. These recommendations will be detailed in the final project report.

### Ethics and data processing

The research will be conducted in accordance with the UK Policy Framework For Health and Social Care Research which sets out the principles of good practice in the management of research^87^. Ethical approval for the study was approved on the 16th July 2021 by the London-Bloomsbury Research Ethics Committee (21/PR/0678). Participants (patients, parents, health professionals and/or other key stakeholders) will be given a participant information sheet at the time of being invited to take part in the study. Prior to any observation or interview taking place, consent will be sought and recorded, either verbally (if the observation/interview is taking place virtually) or in written form (if the observation/interview is taking place face-to-face). For studies 2 and 4, returning a completed survey will be considered implied consent to participate.

Interview data will be digitally recorded, transcribed by a professional transcription company with which a confidentiality agreement is in place. Transcripts will be de-identified and stored along with audio-recordings and de-identified survey responses in the UCL Data Safe Haven which is certified to the ISO27001 information security standard.

### Dissemination

As well as disseminating results through traditional academic forums such as peer-reviewed publications, we will engage directly with health professionals, policy makers, patients and the public. Crucially, we will disseminate our results to the intervention implementers (e.g. NHS England, Genomic Partnership Board), as findings emerge, so any suggested changes to service provision can be considered in a timely manner. Our results will be shared in the form of short reports and/or slide-sets. We will measure the impact of reporting our findings, i.e. any change that have been made as a result of these findings. We will also share regular study updates via the social media channels and newsletters of patient groups including SWAN UK, Genetic Alliance UK and Unique, who are on the advisory team. At the end of the study, we will produce a series of video abstracts aimed at patients and the public to showcase the key findings from the research. We will reach out to those participants that took part in this programme of research, and send them links to these abstracts, so that they can understand the findings from this work.

Anonymised data underlying the results will be hosted in the UCL Data Repository and a DOI will be referenced in research publications.

### Study status

The study has NHS Ethics approval from Bloomsbury Ethics Committee (Rec reference is 21/PR/0678), and data collection has now started.

## Discussion

The NHS GMS will undoubtedly improve the diagnosis and management of patients and their families affected by rare genetic diseases, and provide emotional relief for parents who have been searching for answers. Whilst some of the potential issues (educational, logistical etc) have been identified and are being addressed prior to the start of the Service, there will inevitably be unanticipated barriers and challenges along the way. This research programme provides a unique opportunity to holistically evaluate the expectations and outcomes of the NHS GMS for paediatric rare disease diagnosis, and provide insights and recommendations to improve service delivery. It will also add to our understanding of the experience of parents undergoing and health professionals delivering genomic testing in routine clinical practice.

Our mixed-methods approach will provide rich, comprehensive insights into the facilitators, challenges and barriers of delivering the NHS GMS. Examining both parents’ and health professionals’ experiences will ensure that experiences and outcomes are explored from multiple perspectives. In designing this study, we have engaged with patients as well as other key stakeholders such as health professionals and policy makers at inception to ensure the research will provide important insights for service improvement and to increase the likelihood that the recommendations will be adopted by policy makers. Our advisory team also comprises a broad range of expertise across genomics including geneticists, genetic counsellors, clinical scientists, behavioural scientists, ethicists, health economists and policy makers who can provide critical insight into the study findings and ensure they are fed back to relevant parties in a timely manner. A key challenge for the project is that there are multiple sub-studies that require buy-in from health professionals across a range of specialties. Moreover, the covid pandemic has meant that many research projects are taking longer to get approved and there have been delays in getting the NHS GMS up and running.

In a recent strategy for genomics set out by the Department of Health, the Minister for Innovation wrote that “the biggest gains are being made through collaborations across a range of expertise from clinicians, engineers, social scientists, mathematicians, and data scientists.”^3^. The NHS GMS provides an ideal opportunity to use approaches from social and behavioural science to examine implementation, experiences and outcomes of service providers, patients and other key stakeholders. This work will provide important evidence for both the NHS and other countries implementing genomics into their national healthcare systems.

## Supplementary Material

Full Text XML

Open Peer Review

## Figures and Tables

**Table 1:** Summary of survey measures.

Survey domain	Description	Time 1	Time 2
**Participant characteristics and personality traits**			
Participant characteristics	Child age, parent/carer age, gender, education, number of children, ethnicity, religion and religiosity, income	✓	✓
General anxiety	Generalised Anxiety Disorder Questionnaire (GAD-7). A seven-item measure for screening and severity measuring generalised anxiety disorder. Items are rated on a 4-point Likert scale^73^	✓	✘
Resilience	Brief resilience scale. A six-item measure for assessing the ability to bounce back or recover from stress. Items are rated on a 5-point Likert scale^80^.	✓	✘
Intolerance for Uncertainty	Short version of the Intolerance for Uncertainty scale. A 12-item measure for assessing intolerance for uncertainty. Items are rated on a 5-point Likert scale^79^.	✓	✘
**Attributes of informed decision-making**			
Knowledge	Nine-item knowledge of genome sequencing (KOGS) measure that is context-neutral and focuses on what is involved in having genome sequencing (including ‘what is a genome’), and the limitations and uncertainties of genome sequencing. Each statement is rated as either true, false or don’t know^70^. In addition, we will include a number of knowledge items developed specifically for the this study which relate to the way that the Service is being offered.	✓	✘
Attitude	Five-item scale examining general attitudes to genome sequencing e.g. harmful – beneficial, unimportant – important, measured on a five-point Likert scale^24^.	✓	✓
Self-reported informed decision-making	Question used previously in survey on genome sequencing in the 100,000 Genomes Project^24^.	✓	✘
Decisional conflict	Sixteen-item measure with five-point Likert scale which assess decisional certainty or conflict about a healthcare decision^72^	✓	✘
Decisional-regret	Five-item measure with five-point likert scale which assesses regret or remorse about a healthcare decision, with scores ranging from 0 to 100. DRS scores can be defined into three categories: no decision regret (DRS score 0), mild decision regret (DRS score 1–25), and moderate to high decision regret (DRS score >25)^68^.	✘	✓
Test results	Study specific question to assess what result the patient received (a diagnostic result, a nofindings result or an uncertain result)	✘	✓
**Clinical, psychosocial and behavioural outcomes**			
Parental empowerment	Genomics Outcome Scale: six-item questionnaire with five-point likert scale which captures the theoretical construct of empowerment relating to genomic medicine^74^	✓	✓
Health-related quality of life (child)	EQ-5D-Y (ages 4-15): Comprises five dimensions: mobility, looking after myself, doing usual activities, having pain or discomfort and feeling worried, sad or unhappy. Each dimension has 3 levels: no problems, some problems and a lot of problems. The caregiver (the proxy) is asked to rate the child’s/ adolescent’s health-related quality of life in their (the proxy’s) opinion^75^.	✓	✓
Psychological impact	Adapted 12-item version of the Feelings About genomic Testing Results (FACToR) with five-point Likert scale which measures the specific impact of result disclosure after genomic testing^77^	✘	✓
Family impact	PEDS-QL Family impact module: sixteen-item questionnaire with five-point Likert Scale which explores problems with communication, worry, daily activities, family relationships^76^	✓	✓
Clinical, social and behavioural impact of results	Study specific questions which explore: changes to clinical management, understanding the likely course of the condition, changes to child’s/parent’s lifestyle, connecting with specific rare disease support groups/other families, communication with medical professionals, reproductive decision-making and identification of other at-risk family members. Each item will have 5 levels (not at all – a great deal).	✘	✓
Satisfaction with appointment	Seven-item patient-satisfaction measure for use in a clinical genetics setting^78^	✓	✓

## Data Availability

No data are associated with this article. Figshare UCL: SRQR Checklist for ‘Mixed-methods evaluation of the NHS Genomic Medicine Service for rare diseases: study protocol’, https://doi.org/10.5522/04/16847794 ^88^.

## Plain english summary

### Background and aims

Genome sequencing (where a person’s entire genetic code is mapped) is set to dramatically transform patient care and medical outcomes. Recently, genome sequencing was introduced as part of routine clinical care in the NHS, through the Genomic Medicine Service (GMS). The aim of this research is to understand how genome sequencing is being delivered in the first few years of the Service, in particular what the barriers and enablers are to successful delivery. The focus of the study will be the use of genome sequencing for children with undiagnosed conditions.

### Study design

This is a four-year study in which we will conduct: observations of clinic appointments; interviews with policy makers and health professionals designing and implementing the new service; and surveys/interviews with parents of patients undergoing genomic testing. By the end of this study we will have:

- a better understanding of the intended vs actual outcomes of the GMS,
- insights into what happens during clinical encounters,
- understand what the entire testing process is like for parents from being offered genomic testing to receiving their results and beyond, including the clinical as well as emotional and practical outcomes, and
- understand how healthcare professionals feel about delivering the GMS, particularly those that are non-genetic specialists, including how prepared they feel to deliver genomic testing.

### Patient and public involvement

Parents of children who have been through the testing process have helped us design this study. They have inputted into surveys and topic guides, and will be involved throughout the study as members of the advisory team so that we can ensure the findings are used to improve the quality of care patients and families receive.

### Dissemination

The findings from this research will be shared with organisations such as NHS England and NHS Improvement so that recommendations can be implemented swiftly.
