# Supplementary material for: Mixed-methods evaluation of the NHS Genomic Medicine Service for paediatric rare diseases: study protocol [version 1; peer review: 2 approved, 2 approved with reservations]
Source: NIHR Open Res. Author manuscript; Available in PMC 2022 Jan 28. (PMC7612282)
Supplement: Open Peer Review [file EMS140786-supplement-Open-Peer-Review.pdf]

Open Peer Review

Current Peer Review Status: ? ✓ ✓ ?

Version 1

Reviewer Report 22 December 2021

https://doi.org/10.3310/nihropenres.14352.r28442

© **2021 Krabbenborg L et al.** This is an open access peer review report distributed under the terms of the Creative Commons Attribution License, which permits unrestricted use, distribution, and reproduction in any medium, provided the original work is properly cited.

?

Lotte Krabbenborg
Institute for Science in Society, Radboud University, Nijmegen, The Netherlands
Karine Wendrich
Radboud University, Nijmege, The Netherlands

General:
This article presents the study protocol of a mixed-methods study to evaluate the NHS Genomic Medicine Service for paediatric rare diseases. This is a highly relevant study and we like the mixed-methods approach (surveys, interviews, observations) with the inclusion of different stakeholder groups (designers/implementers, healthcare providers and patients). This way, a variety of important topics and perspectives are covered. The study protocol seems to be very well thought through. However, we would recommend some adaptations to how the protocol is currently presented in the article. According to us, the main points of improvement are a better substantiation of certain decisions and more attention for the broader relevance of this study, as further elaborated in the comments.

Major comments:

1. At the start and the end of the article, we would like this study to be placed in a broader context than the NHS Genomic Medicine Service alone, in order to show the relevance of this study beyond the NHS GMS. For instance, what could other healthcare programs or the field of implementation science gain from your study?
   As an example, in the description of study 1, the authors rightly state:

   "Implementation science, the systematic study of methods that support the application of research findings and other evidence-based knowledge into policy and practice, is increasingly being seen as playing a critical role health services research[47,54]. Previous research on new interventions has highlighted that as well as assessing outcomes, it is valuable to look at the process of the intervention as this can shed light on the mechanisms responsible for whether and how successful it is[55]. This formative work can also enable researchers to suggest ways to answer questions about how interventions might be adapted and respond to change in order to produce positive outcomes[56]."

This kind of information should be presented upfront, already in the Introduction, to highlight the importance of the current study. Similarly, in the Discussion section at the end of the article, the authors only talk about the NHS GMS, whereas this would be a good place to discuss how this study can have a broader contribution, beyond the current study case.

2. The Introduction presents a lot of information. This information is concise and well written, but to us it feels like an overload of information without a clear focus. Related to our previous comment, we suggest the authors to first present some general information about the GM, followed by the relevance and the focus of the current study. Next, the authors could have a “background information” section, which discusses the previous research on topics that are relevant for the study.

3. As the authors mention, the GMS will be implemented for rare diseases and cancer. Why do the authors only focus on paediatric diseases in this study? We would say that it is highly interesting to compare the implementation of the GMS for (paediatric) rare diseases and cancer. So, why has the decision been made to focus on paediatric rare diseases?

Minor comments:

1. When presenting the research approach and conceptual framework, the authors mention that they use a theory-driven approach. We would like the authors to explain why they have chosen these theories. Why are these the most suitable, given the aim of this study? Moreover, the authors could consider to shortly explain the different domains of The Consolidated Framework for Implementation Research and Proctor’s taxonomy, as now it remains quite abstract.

2. The figures are really nice, providing a clear schematic overview of the study and the different sub-studies. In Figures 1 and 2, we recommend the authors to change the titles of studies 3 and 4. Based on the titles in these figures, it is not clear that study 3 is about parents and study 4 is about healthcare providers. As we see it, both studies are mixed methods with parents and healthcare providers respectively. So, our suggestion is to rename study 3 something like: “mixed methods parental perspective” and study 4: “mixed methods medical specialist perspective”.

3. Why are the stakeholder interviews and document analysis in study 1 only performed in year 1 and 4 of the study? We can imagine that adding another timepoint in the middle of the study period would allow for gaining more insight into the implementation dynamics.

4. Regarding the recruitment of patients and healthcare providers in studies 2, 3 and 4: how will you gain access to these patients and healthcare providers? Will this be done through the principal investigator of each of the seven study sites?

5. We really like the idea of a workshop with key stakeholders at the end of the study to further refine the recommendations for practice. Maybe you can include some additional information about this workshop? What kind of stakeholders will participate, how will they be recruited? Will this workshop also be recorded and analysed for research purposes?

6. In study 3, respondents seem to be exposed to a lot of surveys (nicely summarize in Figure 3), which can place a high burden on the respondents. It is our understanding that the main

focus of study 3 is on decisional regret. The authors mention a lot of secondary outcomes. Are all these surveys and secondary outcomes really necessary? You should not just collect as much data as possible, "just because you can", but only in case you really need this information.

7. The authors state: *"Prior to any observation or interview taking place, consent will be sought and recorded, either verbally (if the observation/interview is taking place virtually) or in written form (if the observation/interview is taking place face-to-face)."* - Our experience is that written consent can also be obtained in the case of a virtual observation/interview, for instance by sending the consent form to the participant by e-mail. So, we suggest the authors to also obtain written consent for the virtual observations and interviews.

When addressing these comments, we believe the authors will present a nice article about a highly interesting study protocol. We wish the authors the best of luck with the execution of the study protocol.

Is the rationale for, and objectives of, the study clearly described?

Yes

Is the study design appropriate for the research question?

Yes

Are sufficient details of the methods provided to allow replication by others?

Yes

Are the datasets clearly presented in a useable and accessible format?

Yes

Competing Interests: No competing interests were disclosed.

Reviewer Expertise: Patient participation in research, whole genome / exome sequencing, new medical technologies, ehealth, responsible research and innovation

We confirm that we have read this submission and believe that we have an appropriate level of expertise to confirm that it is of an acceptable scientific standard, however we have significant reservations, as outlined above.

Reviewer Report 07 December 2021

https://doi.org/10.3310/nihropenres.14352.r28439

© **2021 Sperber N.** This is an open access peer review report distributed under the terms of the Creative Commons Attribution License, which permits unrestricted use, distribution, and reproduction in any medium, provided the original work is properly cited.

Nina Sperber

[1] Duke Department of Population Health Sciences, School of Medicine, Duke University, Durham,

NC, USA
[2] Durham VA Health Care System, Durham, NC, USA

The authors have presented a mixed-methods protocol to evaluate implementation of genomic medicine with a focus on paediatric rare diseases. This project includes four parts which will each address different levels of implementation. The first part focuses on a program level, aiming to develop a logic model of the NHS GMS to evaluate concordance between intended and actual outcomes and identify barriers to outcomes by examining documents and interviewing key stakeholders involved in delivering services (including program designers, implementers, and providers). The second part focuses on an inter-personal level by observing providers and families (parents and child patients) conducting genomic testing in clinical settings, using a structured guide used in the 100,000 Genomes Project, to denote observations about appointment structure and provider/patient communication, shedding light on processes and practices in naturalistic environments. The third part will target an individual level, focusing on parents' perspectives about genomic testing with a longitudinal survey and interview data to measure decisional regret, comparing parents who receive a diagnostic result with those who do not. The fourth part will also target an individual level to obtain perspectives of different non-genetic medical specialists about different aspects of implementation including readiness, process, and outcomes using a cross-sectional survey developed with validated measures (acceptability, feasibility, implementation leadership support, and organisational change expectations) and from prior thematic qualitative analysis. Findings from all parts will be integrated to identify overarching findings and recommendations and discussed with key stakeholders.

This paper clearly describes rationale for and objectives of the project. The authors describe a need to understand effects of the relatively newly established Genomic Medicine Service. While some prior work has studied what health care professionals need to integrate genomic medicine into routine care, such as specialized training and access to genomics specialists, gaps exists in our understanding of effective processes and procedures. The authors rationalize their focus on paediatric rare diseases by describing that children make up 50-75% of rare disease cases; they describe a benefit of genomic testing in reducing the "diagnostic odyssey" and need for more information on effects for parents. They outline four objectives commensurate with each of the four parts of the overall design.

The study design is appropriate for the research questions. Generally, they seek to delineate the process of GMS implementation and resultant outcomes, considering different contextual locations and perspectives. Their overall study design of four parts that include multiple types of methods (e.g., document review, individual interviews, participant observations) and mixing of methods (e.g., integrating qualitative and quantitative data about parent-reported experiences or using qualitative data to create a provider survey) will lead them to this end. They have organized the Detailed Study Plan section by research method or technique. For example, they describe the first part (Study 1) as "Implementation interviews with key stakeholders". While the methods seem appropriate for the questions of each study part, the protocol may be strengthened by leading with objective rather than method. For example, the subheading for Study 1 could be "Specify Programme Theory of the GMS" (or something like that). The methods used to achieve that objective would then be described. This change would emphasize the multiple levels of analysis to capture different facets of program implementation and better follow the way it is outlined in the Background and Aims section.

Details of the analysis are clearly described; however, some questions remain:

1. There could be more specificity about how CFIR will be used: it is very large and typically requires discussion ahead of time to prioritize constructs for specific studies, especially for qualitative interviews.

2. More detail is also needed about interviews. How will interview questions be developed? There is a CFIR Guide available for free online. Will those questions be used to ask about CFIR constructs? How long will the interviews last and will they be conducted remotely or in-person? The length of interviews matter because it seems like they could be very long if using CFIR, Proctor, literature, etc. I think more detail in this section would be helpful, including stating if key constructs and outcomes will be selected, rather than asking about all of them.

3. One minor question is whether the sample size of parents in Study 2 for the qualitative interviews will be sufficient to stratify and compare those who receive a diagnostic result vs. those who do not. This comparison was described in the quan. part but not the qual.

4. In Study 3, will recruitment site be factored into modelling? The case was made that they offer diversity. It would be helpful to know how site is accounted for, or rationale for not (e.g., maybe education, ethnicity etc. are proxies?).

5. In Data Synthesis from All Studies section, please describe what you mean by this sentence: “Summary tables will be developed to identify context-specific barriers and facilitators (or suggested changes) to implementation.” It would be helpful to have a better sense of what the final product might look like. It seems that there was emphasis throughout the paper on using explanatory models and identifying mechanisms, and so the final synthesis might include more about process rather than summary of facilitators and barriers. It may be worthwhile to consider Intervention Mapping or something like that.

In general, this is a strong paper with a strong rationale and study design. Changes in formatting would help with clarity. Some additional detail would strengthen description of methods and analysis.

Is the rationale for, and objectives of, the study clearly described?

Yes

Is the study design appropriate for the research question?

Yes

Are sufficient details of the methods provided to allow replication by others?

Yes

Are the datasets clearly presented in a useable and accessible format?

Not applicable

Competing Interests: No competing interests were disclosed.

Reviewer Expertise: Mixed-methods, qualitative methods, implementation science, health services

research, genomic medicine implementation

I confirm that I have read this submission and believe that I have an appropriate level of expertise to confirm that it is of an acceptable scientific standard.

Reviewer Report 07 December 2021

https://doi.org/10.3310/nihropenres.14352.r28440

© 2021 Stark Z. This is an open access peer review report distributed under the terms of the Creative Commons Attribution License, which permits unrestricted use, distribution, and reproduction in any medium, provided the original work is properly cited.

Zornitza Stark
Australian Genomics, Melbourne, Australia

Thank you for the opportunity to review this study protocol.

This will be an important study that will describe the early implementation experience of mainstreaming genomic testing for paediatric rare disease in a public healthcare system. Strengths of the proposed study include the ability to compare against similar data generated while a hybrid research-clinical model was rolled out in the same healthcare system through the 100k genomes project. The study team are also to be commended on their mixed-methods, multi-disciplinary approach to evaluation, and the use of implementation theory frameworks and validated instruments, which will enable comparison with studies from other healthcare systems. Co-design with patients/families/support groups is also a key strength.

Minor reservations:
With regards to Study 1, if I understood correctly, the implementation outcome measures will be gathered through interviews with key designers/implementers of the service. While their insights will be invaluable, consideration should be given to collecting and reporting some objective measures such as uptake and variability of uptake based on geographical location or specialty for example.

Study 2: Clinic observations by a third party can potentially inhibit consultations. I wonder whether consultation recordings, particularly in the era of COVID restrictions and increasing use of telehealth may serve the purpose better?

Study 4: It would have been interesting to collect information on workforce preparedness prospectively rather than retrospectively, or at two time points in order to enable comparison and capture the role of interventions designed to increase preparedness.

Is the rationale for, and objectives of, the study clearly described?
Yes

Is the study design appropriate for the research question?

Yes

Are sufficient details of the methods provided to allow replication by others?
Yes

Are the datasets clearly presented in a useable and accessible format?
Yes

Competing Interests: No competing interests were disclosed.

Reviewer Expertise: Clinical genomics, health economics, implementation science, bioethics

I confirm that I have read this submission and believe that I have an appropriate level of expertise to confirm that it is of an acceptable scientific standard.

Reviewer Report 06 December 2021

https://doi.org/10.3310/nihropenres.14352.r28441

© 2021 Barwell J et al. This is an open access peer review report distributed under the terms of the Creative Commons Attribution License, which permits unrestricted use, distribution, and reproduction in any medium, provided the original work is properly cited.

?

Julian Barwell
Leicester Royal Infirmary, University Hospitals of Leicester NHS Trust, Leicester, UK
Maurice Dungey
University Hospitals of Leicester, Leicester, UK

From Dr Julian Barwell
This is a very welcome addition to the portfolio to assess the impact of genomic medicine. I am pleased to see a mixed model focus on the patient experience from expectation through to result delivery and beyond. The team assembled is very strong.

In order to make this review as insightful and impactful as possible I believe it would be helpful to use the staff review work package to ask a number of targeted questions:

1. Technical support: Technically do the clinicians and scientists feel that the appropriate infrastructure is in place to assist with consent, sample collection, logistically with the tracking of samples and delivery of genomic results through interpretation of variant pipelines, result placement in electronic patient records and governance of result interpretation and patient management taking into consideration variants and the presence or absence of family history/clear dysmorphological features?

2. Infrastructural support: Did the support of Clinical Research Network help when this became available and what barriers were felt locally when requesting managerial support given acute and social care strains on hospital finances? Has this support been maintained in between projects and new downstream investment funding?

3. Diagnostic decision making: Do the clinicians and scientists feel that the appropriate genetic and genomic tests are being ordered to ask the clinical question being asked with regards to sensitivity and specificity? For example taking into consideration read depth and other mechanisms of disease such as copy number variants, uniparental disorder or trinucleotide repeats.

4. Clinical Impact: Has an appropriate downstream cost-benefit impact analysis been carried out on appointments and investigations versus screening, personalised treatments and reproductive health decision making on the basis of the identification of additional diagnostic results?

5. What impact has the introduction of genomic medicine had on the care delivered by clinicians beyond the early adopters that have led or assisted with recruitment of patients to flagship implementation projects.

6. Views on next steps to break down barriers to implementation to mainstreaming and the use of personalised medicine more broadly such as the role of pharmacogenetics, tumour or microbial sequencing and polygenic risk scores?

With assurance that these areas will be covered I would be very happy to support this protocol.

From Dr Dungey

Thank you for the opportunity to review ‘Mixed-methods evaluation of the NHS Genomic Medicine Service for paediatric rare diseases: study protocol’. As the authors point out Whole-Genome sequencing is on the cusp of becoming increasingly available, utilised and mainstreamed as part of the NHS England Genomic Medicine Service (GMS). The enthusiasm, preparedness, impact and consequences of this on healthcare workers including genetic specialists, and particularly those working in medical specialties who may order tests in the mainstreamed setting, is currently unknown. For patients (and parents of affected children) it is currently uncertain what their attitude and understanding of genome sequencing will be; and therefore the patient experience and consequences of GMS is unclear.

This mixed-measures project encompasses four studies with four key aims (as laid out in the protocol):

1. Identify the resources, activities and intended and actual outcomes of the NHS GMS; identify any potential barriers to achieving the intended outcomes during the early years of the Service (2022–25);

2. Understand the processes and practices taking place by examining the interactions between health professionals and parents/patients during pre-test counselling and results delivery appointments;

3. Examine the experiences and outcomes of genomic testing that parents report over time;

4. Identify the preparedness and experiences of medical specialists involved in delivering genomic medicine in mainstream NHS care in the first few years of the Service, and identify elements which make this easier or more difficult.

There are a numerous strengths to these studies. The protocol is grounded in theory and utilises a number of different established frameworks to assess their aims. The multiple studies with different designs and involving varied stakeholders will give an interesting full picture of the impact of implementing GMS and, some insights of how to improve implementation for other services. Another big strength is the involvement of key stakeholders in the study planning and continued involvement throughout the studies and in dissemination of their findings. Studies 3 and 4 use mixed-measures approaches that will give valuable information on patient (or specifically parent) experiences and decisional regret, and the experiences of mainstreamed genomic testing.

There are a number of challenges in the study designs:

- Recruitment to the study is crucial. The study uses purposive sampling, which is a valid qualitative approach but may have a number of weaknesses because the results are designed to be immediately clinically applicable, and not just theory generating. Potential concerns would be the a reliance on the researchers judgement on correct participants to approach, the risk of bias, and whether the results are less generalisable.

- An explanation of how the researchers will involve the key stakeholders around their clinical responsibility has not been documented, and how they will account for bias if certain groups are under/over-represented in the study.

- In observing clinical interactions, it is possible that observation will alter both the health professional and the parent/patient's actions. This is a considerable weakness of observation and must be acknowledged in interpretation of results.

- In the second study the authors will attempt to interview participants from a variety of settings (different sites, conditions, results, ethnicities, socioeconomic backgrounds, type of healthcare professional). This will likely make results harder to interpret and with only 40 interviews the data collected may be too heterogenous.

- The authors plan to disseminate the findings of their work to the stakeholders throughout the study so that the service provision can be improved, it is unclear how this will be quantified in terms of changes made and the impact of these changes.

- It is unclear from the protocol the details of who (and how many researchers) will be conducting interviews, observations, and data analysis. These details need to be clarified.

- It is important there is transparency of how decisions are made in the data synthesis and interpretation from all studies and how recommendations are developed.

Overall, this appears to be a thorough research project that will give recommendations for other services. On the whole the methodology is sound, and we are happy to approve with some considerations to the above points.

Is the rationale for, and objectives of, the study clearly described?

Yes

Is the study design appropriate for the research question?

Partly

Are sufficient details of the methods provided to allow replication by others?
Partly

Are the datasets clearly presented in a useable and accessible format?
Yes

Competing Interests: Dr Barwell: Clinical Research network Division 3 Clinical lead for the East Midlands Advisor to AstraZeneca Engagement Director to Global gene Corp None for Dr Dungey

Reviewer Expertise: Genomic Medicine

We confirm that we have read this submission and believe that we have an appropriate level of expertise to confirm that it is of an acceptable scientific standard, however we have significant reservations, as outlined above.
